# Roll-out of Medical Male circumcision (MMC) for HIV prevention in non-circumcising communities of Northern Uganda

**DOI:** 10.11604/pamj.2013.15.100.2338

**Published:** 2013-07-15

**Authors:** David Lagoro Kitara, Andrew Ocero, Joan Lanyero, Felix Ocom

**Affiliations:** 1 Faculty of Medicine, Department of Surgery, Gulu University, P.O Box 166, Gulu, Uganda; 2 Northern Uganda Malaria and Tuberculosis (NUMAT), P.0. Box 946, Gulu, Uganda; 3 Northern Uganda Health Integration to Improve Services (NU HITES), Gulu, Uganda

**Keywords:** Medical Male circumcision, Northern Uganda, scale-up of services, non-circumcising communities

## Abstract

**Introduction:**

Recent studies have shown that circumcision reduces HIV/AIDS infection rates by 60% among heterosexual African men. Public health officials are arguing that circumcision of men should be a key weapon in the fight of HIV/AIDS in Africa. Experts estimate that more than 3 million lives could be saved in sub-Saharan Africa alone if the procedure becomes widely used. Some communities in Uganda have misconceptions to MMC and resist the practice.

**Methods:**

To roll out MMC to a non-circumcising population of Northern Uganda from June 2011 as a strategy to increase access and prevent the spread of HIV/AIDS.

**Results:**

Circumcision in a non-circumcising communities of Lango and Acholi sub-regions with a population of about 0.5 million mature males 15-49 years. Enrolment was voluntary, clinical officers, nurses carried out MMC after training in the surgical procedure. Mass sensitization and mobilization was conducted through radios, community leaderships and spouses. Cervical cancer screening was incorporated at circumcision sites and used as incentive for the women. Circumcisions were conducted at static sites, camps and outreach services where VCT and adverse events (AEs) were recorded and managed. All clients assented/or consented.

**Conclusion:**

A total of 26, 150 males were circumcised in eight months. The AEs rate was 1.2% and was mild. 2,650 women were screened for cervical cancer and positive test rate was 1.7%. Mobilization and sensitization were by radios and spouses’ involvement in cervical cancer screening exercise.

## Introduction

Male circumcision is the surgical removal of some or all of the foreskin (or prepuce) from the penis [1]. Several types of researches have documented that male circumcision significantly reduces the risk of HIV acquisition by men during penile-vaginal sex. Compared with the dry external skin surface, the inner mucosa of the foreskin has less keratinization (deposition of fibrous protein), a higher density of target cells for HIV infection (Langerhans cells), and is more susceptible to HIV infection than other penile tissue in laboratory studies [2]. The foreskin may also have greater susceptibility to traumatic epithelial disruptions (tears) during intercourse, providing a portal of entry for pathogens, including HIV/AIDS [3]. In addition, the microenvironment in the preputial sac between the un-retracted foreskin and the glans penis may be conducive to viral survival [1–3]. Finally, the higher rates of sexually transmitted genital ulcerative disease, such as syphilis, observed in uncircumcised men may also increase susceptibility to HIV infection [4, 5–7].

Three randomized controlled clinical trials were conducted in Africa to determine whether circumcision of adult males would reduce their risk for HIV infection [8]. The study conducted in South Africa [9] was stopped in 2005, and those in Kenya [10] and Uganda [11] were stopped in 2006 after interim analyses found a statistically significant reduction in male participants’ risk for HIV infection from medical circumcision [8, 9–13].

In these studies, men who had been randomly assigned to the circumcision group had a 60% (South Africa), 53% (Kenya), and 51% (Uganda) lower incidence of HIV infection compared with men assigned to the wait-list group to be circumcised at the end of the study [8, 9, 10, 11]. In all three studies, a few men who had been assigned to be circumcised did not undergo the procedure, and vice versa. When the data were reanalyzed to account for these occurrences, men who had been circumcised had a 76% (South Africa), 60% (Kenya), and 55% (Uganda) reduction in risk for HIV infection compared with those who were not circumcised [8–10].

Lack of male circumcision has also been associated with sexually transmitted genital ulcer disease and chlamydia, infant urinary tract infections, penile cancer, and cervical cancer in female partners of uncircumcised men [1, 8, 12, 13]. The latter two conditions are related to human papilloma-virus (HPV) infection. Transmission of this virus is also associated with lack of male circumcision which was observed in a recent meta-analysis including 26 studies that assessed the association between male circumcision and risk for genital ulcer disease [8, 12, 13]. The analysis concluded that there was a significantly lower risk for syphilis and chancroid among circumcised men, whereas the reduced risk of herpes simplex virus type 2 infections had a borderline statistical significance [4, 8, 12].

Reported complication rates depend on the type of study (e.g., chart review vs. prospective study), setting (medical vs. non-medical facility), person operating (traditional vs. medical practitioner), patient age (infant vs. adult), and surgical technique or instrument used [1, 8, 12]. In large studies of infant circumcision in the United States, reported inpatient complication rates range from 0.2% to 2.0% [1, 8, 12, 14, 15]. The most common complications in the United States were minor bleeding and local infection. Similarly, in the recently completed African trials of adult circumcision, the rates of adverse events possibly, probably, or definitely attributable to circumcision ranged from 2% to 8% [8, 12]. The most commonly reported complications were pain or mild bleeding. There were no reported deaths or long-term sequelae documented [9, 10, 11, 16].

This community where this research was being implemented is a Luo community which is a non-circumcising community. A recent survey in Uganda indicated that northern region had the lowest prevalence of circumcised person of all regions with a rate of only 2.4% [17, 18]. This community resists circumcision and has a derogative local name, “Layom” which literally means “glans exposed like a penis of monkey/baboon”. Traditionally in the Acholi and Lango community, a man known to have no foreskin would be denied courtship/married to the village/community because it was considered an abomination/curse and thus would transfer unfavourable/unwanted genes to the population if allowed to marry a woman. Local songs would be composed about such individuals so that no woman would even attempt to court him. The person would be stigmatized in public and kept out as an outcast of the community. He would not be allowed to participate in community activities such as group hunting, bathing, farming, politics and community leadership because he would be considered a bad omen to the community. Similarly, a study conducted among the Luo of Kenya indicated that some members of the community were in fear of a possibility of discrimination and would shun recently circumcised men, especially when community members were older and/or less educated [19].

**Objective:** To increase uptake of MMC among males 15-49 years in a traditionally non-circumcising communities of northern Uganda in order to support HIV/AIDS/ STI prevention & spread strategy as recommended by the Uganda Ministry of Health.

## Methods

**Study design:** We conducted a prospective interventional study on traditionally non-circumcising communities of Northern Uganda between June 2011 and January 2012.

**Study site:** This study was conducted in Acholi and Lango sub-regions of Northern Uganda. This region is just recovering from the 20 year old civil war. The region is strategically located and endowed with transport terminals with pivoted role in a vast and profitable distribution of goods. The population is largely rural, many of whom were displaced into Internally Displaced Peoples camps (IDPS) for safety from the insurgency. According to the Uganda Bureau of Statistics [18], the region has a population of about 3.5 million people with the female constituting about 52% of the total population. Majority (over 65%) of the population in the region are young people of less than 20 years [18].

**Interventional Population:** The focus of the circumcision was among the sexually active group between the ages of 15-49 years. These were the target group and were estimated to be approximately 0.5 million males in both sub-regions (Table 1).

**Table 1:** Shows the circumcision targets for the 2 sub-regions set at the planning phase of the project

Indicators		Targets
Number of individuals provided with safe male circumcision		26,000
Individuals reached through camps	Per camp	200
	Monthly	3,200
	**Total**	16, 000
Individuals reached through health facility-conducted outreaches	Per outreach	30
	Monthly	990
	**Total**	4,950
Individuals reached through health facility-static sites	Per Health center	40
	Per hospital	80
	Monthly (16x40+5x80	1,040
	**Total**	5,200
**Grand total**		26,150

**Framework:** Policy and communication strategy for roll-out for circumcision was adopted from the Uganda Ministry of health guidelines of 2010. Re-orientation of key staffs in large scale circumcision management and training of clinical officers, nurses and theatre staffs on the additional responsibilities of caring for and follow up of circumcised persons and prevention of complications was conducted. Sensitization and mobilization of the communities through mass media (radios), messages through community structures, youth groups, churches and traditional leaderships was performed. Expert clients were trained to mobilize and sensitize communities on benefits and usefulness of circumcision. Spouses were pillars for the mobilization efforts and Cervical cancer screening services for women was included as part of the service provision during the project implementation.

Couple counseling and testing for HIV/AIDS, circumcision for the men, cervical cancer screening for women at the same visits and condom distribution were used as motivators for the teams. Six weeks of abstinence in postoperative period was recommended to clients at the counseling sessions. Circumcisions were conducted through circumcision camps, outreaches and static services in health centers in the region.

**Data Collection:** Data was collected through structured questionnaire which was designed to capture the socio-demographic characteristics, pre and postoperative findings, VCT results, type of surgical procedure, adverse events (AEs)/postoperative complications. Postoperative review was conducted on 2^nd^ and 7^th^ postoperative days. All clients with AEs were classified and managed according to protocol.

**Surgical kits:** Pre-packed circumcision kits were supplied by USAID; the funding agency to Northern Uganda Malaria and Tuberculosis program (NUMAT); each kit consisted of disposable instruments and sundries packed to be used on a client. Local anesthetic agents; Lignocaine 1% (3mg/kg) and Bupivacaine 0.25% (o.5mg/Kg) was mixed in a ratio of 1:1 and administered by nerve root block (Using a fine needle (23 gauge) to inject 1-2 ml in base of penis at 11 and 1 o'clock positions and inject 1 ml of local anesthetic laterally towards ventral surface to complete a ring at base of penis) or ring block (inject eutectic mixture for bupivacaine & lignocaine) subcutaneously around the base of the penis to produce a ring block and thus block the cutaneous nerves from the scrotum and circumcision surgeon had to wait for 3-5 minutes for the anesthetic agent to take. Forceps guided surgical procedure was the most commonly used circumcision technique.

**Ethical consideration:** Consent/Assent was obtained from each client and confidentiality was ensured throughout the study and intervention. Ethical approval was obtained from the Uganda National council of Science and Technology (UNCS&T).

**Data analysis:** Statistical Package SPSS version 12.0 was used to analyze the data. Descriptive statistics were obtained for these variables.

## Results

A total of 26,150 males were circumcised over an eight months period in multiple health centers in Northern Uganda. The mean age was 19 years (SD 4.8), range 15-59 years, median 19 years. The adverse events rate was 1.2% which was mild and easily correctable. Mild bleeding and hematoma were the commonest AEs. Those HIV/AIDS positive males were counseled and referred for further care. A total of 2,650 women were screened for cervical cancer and with a positive test rate of 1.7%. The mean age of the women screened was 29 years (SD 5.6), range 16-48 years, median of 30 years. All women who tested positive for cervical cancer were referred to the regional hospitals for further management. Mass mobilization and sensitization were conducted mainly through the local FM radios talk shows, Disco Jocker (DJ) jingles and announcements. Expert clients were identified and trained to delivery correct MMC messages; youth groups and youth councils were involved in dialogue and facilitated to conduct community meetings; elders and traditional leaders were engaged in public discussions; church leaders and opinion leaders were trained and engaged in circumcision discussions; spouses’ involvement in cervical cancer screening exercises were encouraged to present the benefits and usefulness of circumcision to the population.

**Figure 1:**
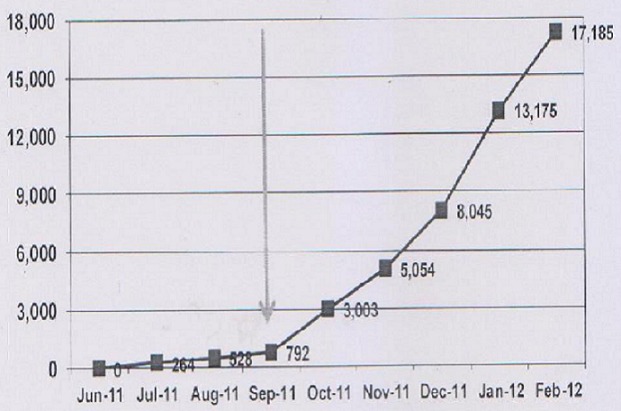
Community's response to circumcision after scaling up of MMC activities as shown by the arrow marking the introduction of intense mass mobilization and cervical screening exercises for women

Overwhelming turn up and demand for circumcision was created in this population by this approach (over 26,800 turned up thus nearly overwhelming the system) (Figure 1). Couples turned up in large numbers for circumcision and cervical cancer screening exercises (10.13%) and compliance to the postoperative care was improved and we noticed very few complications reported (only 1.2%). Women who were found with suspected cervical cancer lesions were referred for further management to referral hospitals.

**Adverse Events (AEs):** The AEs were all mild and easily correctable; they were mostly active bleeding and hematoma formation. The majority (290/314) occurred within the first 48 hours and reported in the morning of the 1^st^ postoperative day. One severe adverse event occurred when a client with a bleeding disorder was not detected at history taking and physical examination. He was later transferred to a regional hospital where he was successfully managed by an expert team; bleeding was controlled and client was discharged within one week of hospitalization. Postoperative sepsis was observed in less than 0.5% and mainly at suture lines. Postoperative erections were common among the teen agers at the morning of the 1^st^ three postoperative days and these were managed by administration of paracetamol (1000mg) and counseling.

## Discussion

Luo people (Acholi and Lango) are among the few Ugandan tribes that do not traditionally circumcise their males as an initiation to manhood [18, 20]. The elders believe that circumcision robs manhood. Instead of circumcision, some researchers have argued that some of the Luo traditionally welcomed their young men and women into adulthood by whacking out six front teeth [20]. Reports from the Uganda Bureau of Statistics indicate that most Luo in Uganda are mainly Christians, although many still uphold most of their traditional cultural customs [18]. This is especially true for those living in the rural areas [18, 20]. However, some of the Luo cultural practices are now regarded as retrogressive and are slowly fading away, such as wife inheritance [20]. The tradition, customs and religious beliefs among this community significantly contribute to their beliefs, attitudes, and practices to circumcision. There are ranges of misconceptions to circumcision among these communities e.g. circumcision is painful and deprives one of his vital body parts; it exposes the glands penis making one appear like a baboon; it makes one unable to conduct routine work; circumcision makes a man to wear a skirt or wrap up in a towel; circumcision converts a Christian to Islam and many other issues that have tended to give a negative publicity to circumcision among the community [19]. A study conducted in western Kenya among the Luo population indicated that the commonest identified barriers to MMC were pain, culture and religion, cost, possible adverse events (AEs), and the potential for risk compensation (i.e., an increase in risky sexual behavior following MC) [19, 20].

Traditionally in some of communities in this region, a male born without/retracted foreskin would be stigmatized [19]. Local songs would be composed in their names describing how their glans penises were exposed like that of a baboon. Every effort would be made by the community to prevent such a male from courting/marrying any female in the neighbor-hood/community. They believed such a person would transfer unwanted genes to the population. Circumcised penis was considered a curse from God and such a male would not be allowed to participate with other community members in join community activities such as group faming, hunting, bathing and community leadership. A similar study conducted among Luo of Kenya (kin brothers to the Acholi and Lango) indicated that some members of the community were in fear of a possibility of discrimination and were thought to shun recently circumcised men, especially when community members were older and/or less educated [19].

In order for this project to succeed, we had to organize ourselves and counter the misconceptions of the community through the most reliable means of communication- the mass media [20–22]. Reliable and prominent leaders became advocates of circumcision and provided information in a way that would not injure the communities’ traditional perceptions to circumcision. Westercamp and Bailey reviewed 13 MMC acceptability studies and concluded that the studies were consistent in identifying certain factors that facilitated MMC uptake; including the beliefs that MMC lead to improved hygiene, protection from sexually transmitted infections (STIs) and HIV, improved sexual pleasure and performance, and greater acceptability by other ethnic groups [19]. We promoted messages that would improve the image of MMC in this community. Reiss et al. reported that recently circumcised men said they were able to perform more rounds of sex; they were able to use condoms more easily; and they sustained fewer cuts on their penis during sex [20].

**Mass sensitization and mobilization:** Mass sensitization and mobilization was achieved by using FM radios which was crucial for the success of the project. Previous studies in northern Uganda had indicated that 95% of the health messages reached the communities through the various FM radio stations [21]. We exploited this by using the radio talk shows, announcements and DJ jingles for delivering circumcision messages [22]. Expert clients, youth leaders, elders and religious leaders were engaged in delivering health messages on mass MMC. Westercamp and Bailey in their study recommended engagement of religious leaders before promoting MMC in a country [19]. This similarly became an important recommendation in scaling-up of MMC services in this region.

The introduction of cervical cancer screening services for the women brought women to participate in the project. They in turn encouraged their husbands to accept circumcision and adhere to the postoperative guidelines. Majority of married clients who turned up indicated that they were encouraged by their wives who had undertaken cervical cancer screening at the circumcision sites. Previous studies had observed that females had the best response to ill-health and the best health seeking behaviours in Uganda more especially among the Acholi people [21–23]. A satisfied client brought in the next client and thus the whole community became involved in the campaign. A pain free postoperative period because of the eutectic local anesthetic used was crucial for the propagation and promotion of circumcision project in this community.

**VCT and HIV prevalence:** Participants were young men mostly in their late teens. The HIV prevalence was 5.7%, which was much lower than the 8.2% for the general population of Northern Uganda [17, 18]. This could have been because of the age bracket, young single adolescents and mostly in secondary schools were the majority. However, this rate was relatively higher than the rates in the same age group in other parts of the Uganda [17, 18, 23]. This may perhaps be a result of over 20 years of insurgency where there has been moral decay and a high prevalence of HIV/AIDS in the population [22].

**Control of pain:** The use of eutectic combination of lignocaine and bupivacaine and proper administration ensured that there was a pain free period during and immediately after surgery. Paracetamol was administered orally for the postoperative pain after the weary of the local anesthetic. The combination of these 2 agents helped to calm the community that had believed that circumcision was always painful. Boda boda riders returned to their routine work and shared their experiences with their colleagues and customers who equally propagated the MMC messages to the community. Studies conducted in Nyanza Province reported that the primary reasons men chose circumcision were to enhance protection from HIV and STIs, improve hygiene, decrease risk of penile cancer, and improve sexual satisfaction for men and their sex partners; while the primary reasons that men chose not to be circumcised were pain during/after the procedure, long healing period, AEs, culture or religion, and time away from work [24–27]. The control of pain became a major driver to acceptance of circumcision among this population.

**Adverse events:** We recorded a 1.2% adverse events rate which was comparable to most experiences in other studies [24–27]. All events were mild and were corrected within 48 hours of the postoperatively except one client with a bleeding disorder who required 1 week of hospitalization. Postoperative circumcision reviews were conducted on the 2^nd^ and 7^th^ days. Most teen agers reported early morning erections on the first three postoperative days which were controlled by counseling and administration of paracetamol orally.

### Lessons Learned

In order to improve access to MMC activities in a traditionally non-circumcising community, the following could be the most effective way to achieve it:

1. Conduct mass campaigns using the most reliable means and media of communication: The campaigns should be short term, of intensified nature and should be performed to accelerate the numbers of males to be circumcised.
2. Implementing Forceps guided procedure in a safe and timely manner enhanced efficiencies and increased numbers circumcised per site.
3. Coordination of various organizations’ activities so that correct and consistent messages is circulated to the population indicating that circumcision would be delivered safely and efficiently. This involved political leadership, health administrators and the public so as to build momentum of the public in normalization of MMC activities. Engaging women in service such as cervical cancer screening exercises was key to the success of the project. Synergies such as reaching out to males through HIV testing and female cervical cancer screening should be encouraged and promoted
4. Taking services nearer to the population by organizing outreaches and circumcision camps made many community members access these services.
5. Regular circumcision kits supply and proper equipment management. The forecasting procurement and management of infectious waste generated by the MMC program were essential for the scale-up. Good planning and coordination between the implementing partners to ensure that necessary supplies are available and that hazardous wastes were safely disposed.

## Conclusion

MMC can be scaled-up in a non-circumcising community using intense mass sensitization and mobilization using the media and communities’ leadership structures. There is need to increase sustainable activities that can increase MMC uptake in this non-circumcising communities of Northern Uganda including among other things dispelling MMC misconceptions; increasing involvement of religious leaders, women's groups, and peer mobilizers for MMC promotion; and increasing the relevance of MMC among men who are already practicing HIV prevention methods. Pre-packed circumcision kits should be made readily available and prevention of AEs could increase further the uptake of MMC in the region.
